# Abstracts and Gleanings

**Published:** 1880-05-20

**Authors:** 


**Sims' Speculum always at Hand.**—The Chicago Medical Gazette gives the following valuable piece of gynæcological information. The index and middle finger of the right hand may be used as a perineal retractor in place of the ordinary Sims' speculum. They may be introduced with the patient in Sims' latero-prone position, the operator standing back of the patient, on the side of the table, in exactly the position of the assistant who holds the speculum in the ordinary way. In this manner the cervix and vagina may be exposed almost as well as by the speculum. This method of exposing the parts may be of great use when a speculum is needed and not accessible, in the application, for instance, of the tampon in sudden cases of hæmorrhage, or in consultations at a distance, when for reasons not anticipated, it becomes necessary to examine the pelvic organs.

### ABSTRACTS AND GLEANINGS.

**Ringworm of the Scalp.**—In regard to the treatment of ringworm of the scalp every one will find disappointments enough. Some cases yield very easily, while others are most stubborn, even under judicious and intelligent treatment. Although a local disease, internal measures should never be neglected, for there is good reason to believe that the parasite will not attack, or will not remain, on a perfectly healthy skin; there is always debility, certainly in cases which prove rebellious to well-devised local measures.

It is well to remember that the lower forms of vegetation—mosses, mould, etc.—do not grow and flourish on healthy trees with good soil and plenty of sunlight, but on decaying matter, and in dark, damp places.

The actual internal treatment must depend a good deal on the individual affected, and each case should be specially studied, and all elements of ill health and debility removed. Arsenic may be of benefit, but it is very doubtful if it is of much service when given in a routine manner and alone.

The local measures must also vary with the case. Thus, in the inflamed spots of tinea kerion, an active anti-parasitic treatment, which might be very valuable in another case, would prove disastrous. In this form I have usually employed the most soothing remedies until the inflammation has quite subsided. Thus sweet almond oil will suffice to cause the subsidence of the inflammatory masses; or a valuable preparation is the mixture of oil of cade and cod-liver oil, in the proportion of one to four, or thereabouts. Zinc or bismuth ointment, half a drachm to the ounce, has often rendered me very good service.

The hair should always be cut short all over the head at the beginning of the treatment of ringworm of the scalp in children. If the disease is very extensive it is better to have it shaved at once with the razor. This is in order that the treatment may be better carried out, and that each and every spot of disease may be discovered. In earlier days I first tried to do without cropping the hair, but generally was obliged to resort to this in the end.

My greatest reliance has been upon sulphurous acid, as explained in the preceding article, though, of course, like any other remedy, it is not universally successful. It should be very thoroughly applied (rubbed in) morning and night, and some ointment applied at bedtime. Of ointments, that of the yellow sulphate of mercury, turpeth mineral, fifteen to thirty grains in the ounce, is very effectual, though it sometimes causes considerable inflammation. A very good ointment is made with a drachm of oil of cade, one or two drachms of the ointment of the red oxide of mercury, and six of rose ointment.

Many other local measures may be found in the books, most of which are of value. Many use the tincture of iodine, but I have found it to fail so repeatedly, that I seldom employ it. The liquor iodinii compositus is a more effective remedy. The oleate of mercury, in five or ten per cent. solution, is a tolerably clean remedy, but has not been as effective in my hands as I had hoped. When used very freely, I

have seen salivation in one case of ringworm of the scalp. Bichloride of mercury, in weak solution, two to five grains to the ounce, is also cleanly, and recommended by some. But I fear to place it in every patient's hands lest poisonous results should follow, as such have been reported to have occurred. I have only used it as I applied it myself after epilation.

Epilation is frequently necessary, though it is a measure which is with difficulty carried out in this disease, parents seldom appreciating the necessity. I have occasionally been obliged to resort to it, and in one instance of a little girl, 7 years old, Agnes ——, the mother and I pulled out thirty-three thousand eight hundred and fifty hairs before the disease was cured.

Much difficulty is sometimes experienced in accomplishing the extraction of the hairs, owing to their brittleness, resulting from the penetration of the parasite into the hair; but after pulling for a while the hairs will be found to be more firm, and can be removed entire. Sometimes the hairs break off but little, the reason of which is not apparent.

A word may be added in reference to the subject of over-treated cases of ringworm. While managing the cases in the public institution referred to, cases would continually be observed where the treatment itself gave rise to considerable general pustular inflammation of the scalp, and in other cases a very scaly condition would be induced, which had nothing to do with the original disease. This I have sometimes observed also in private practice.

In these cases it was necessary to abstain from all treatment for a while, except such of a very soothing character, and, allowing the artificial eruption to subside, to watch for the return of the parasitic affection. After resting thus for a while the cases were left entirely alone for a few days, and then the hairs and scales carefully examined with the microscope, when it would be fonnd that there was no remnant of the former trouble. In some cases, however, the parasitic disease was found to be still present, and the appropriate treatment was resumed.

The prognosis of ringworm of the scalp should always be guarded, for though in the abstract it is a perfectly curable disease, still in actual practice there are so many exigencies which may arise, that it can never be predicted with certainty what will be the progress or result in any particular case or cases. It rests largely with the patient and friends.—*Archives of Dermatology*.

**Painless Cure of Internal Hemorrhoids.**—Dr. R. A. Vance, in Med. and Surg. Reporter, says:

In the form of internal hemorrhoids called "capillary" there may simply be hyperæmia and thickening of the mucous membrane. It is this variety of the affection which so readily yields to the application of nitric acid. In the "arterial" hemorrhoid there is one or more tumors, with hypertrophy of the submucous cellular tissue; the name of the variety is due to the fact that large arterial trunks may be traced into the growths. This form of tumor yields readily to the treatment soon to be described. The "venous" hemorrhoid is a later stage of the arterial; the arterial trunks are no longer so prominent, but the hypertrophied submucous cellular tissue still remains. These tumors are the most common variety of internal hemorrhoids the practitioner encounters.

An enema of warm water, or water not too hot to be readily borne, suffices for the complete exposure of all forms of internal hemorrhoids. In order to understand the painless character of the treatment now advised, the practitioner should patiently investigate the sensibility of numerous cases of internal hemorrhoidal tumors. Inject as much warm water as the patient can stand; then, as soon as the enema is voided and the lower part of the rectum everted, exposing the hemorrhoidal tumors, carefully study the sensibility of the rectum about the tumors, the base of the tumors, and, finally, the summits of the tumors. As a general rule, when the tumors are uninflamed, the most sensitive part is a narrow band just at the base of the growth, where the lining membrane of the rectum is reflected on the hemorrhoid. This band may not be the tenth part of an inch in width. The rectum walls just beyond this band are slightly more sensitive than the general surface of the rectum in the neighborhood. Tracing the sensibility of the tumor itself from the band encircling it at its junction with the rectum to its summit, it will be found that there is rapid loss of all perception of painful impressions as you pass from base to summit, until finally, at the top of the tumor, a needle can be run through its apex without exciting but little if any pain. This anæsthetic region at the top of the tumor varies in different cases; as the outlet of the rectum is neared, and more or less integument enters into the formation of the tumor, it grows smaller and smaller; on the contrary, the deeper they are situated the larger it becomes. In every tumor situated within the line marking the junction of skin and mucous membrane, a region of greater or less extent in which a needle can be passed without exciting but very little pain can be found, if sought for intelligently.

The recognition of this important fact is no discovery of my own. Dr. Bodenhamer, of New York City, described certain experiments which satisfied him that the mucous membrane covering the tumor was much less sensitive than that lining the rectum, and published the same in the appendix to his work on "The Physical Exploration of the Rectum," which appeared in 1870. As a result of my own investigations in this line, I can state that in certain cases there is an absolute loss of all sensibility to pain at the summit of internal hemorrhoidal tumors; that in all, the most sensitive part is the band I have described; and that in no case is the mucous covering of any other part of the tumor as susceptible to painful impressions as the normal lining of the rectum.

If a seton be passed through a swelling due to a collection of inflammatory products, the nutritive processes are so modified that disassimilation and absorption are increased. In January, 1878, a gentleman came to me for advice, who suffered from four large hemorrhoidal tumors. In the largest of these growths there was complete anesthesia of the summit, and I passed a needle with a double thread through the top, with the intention of strangulating the apex of the tumor. At this point he interrupted me to say that he could not be away from his business for a day that month; that, therefore, I must do nothing that would require him to lay up. Rather than withdraw the ligature, I cut loose the needle and tied the thread, leaving a loop about an inch long; there was not the sixth of an inch between the point where the needle entered the tumor and where it emerged. Five weeks subsequently the same gentleman returned; a little parchment-like thickening alone marked the site of the tumor into the summit of which I inserted the seton. The gentleman declared he had no more than the usual amount of discomfort with his rectum; that he was not conscious of anything unusual taking place there; yet the tumor had disappeared. I explained matters to him, and inserted two setons—one in each of the two purely internal hemorrhoids remaining—leaving one tumor on the border of the anus and rectum untouched. Through some misunderstanding I did not see him again for a fortnight; both tumors were then markedly diminished. In the end the setons sloughed out, and both tumors were cured. The remaining tumor I have since excised, its situation at the junction of rectum and anus preventing the employment of either seton or ligature.

Since my experience with this case, I have had numeraus opportunities of testing this method of treatment. It is only applicable in hemorrhoidal tumors which are not inflamed. The tumors, also, must be purely internal tumors; the deeper and larger, the better. Again, there is nothing to prevent the patient continuing his ordinary avocation during treatment. Finally, I am led to believe that this is the method employed by certain irregular practitioners who have great reputations in rectum surgery; that they pass a seton in those cases of internal hemorrhoids that they undertake to cure "without pain or detention from business," and, of course, "without caustic or the knife."

In passing the setons it is necessary that the tumors be completely extruded. This done, by means of enemata of hot water, investigate the condition of the uppermost tumor; find the spot where sensibility is least, and pass a curved needle through the summit, being careful not to go too deep, or to bring the needle out too far from where it is entered. By attending to these points, the needle is passed without pain; yet, if passed too deep, or carried too far from the entrance, not only will pain be excited, but the rectum will contract and the tumors return. As soon as the needle is passed tie the ligature into a loop about six inches long; this loop will enable the surgeon to control the movements of the whole mass of tumors. Next, pass a ligature through each of the other tumors, making the threads double, and tying them so that there is not more than an inch of loop in all. Finally draw down the upper tumor, by means of the double thread through it, and tie a knot in the latter, so close to the tumor that all the setons may be alike in length; then cut off the superfluous thread and return the tumor within the anus. This done, the patient should be instructed to keep his bowels freely open, daily, but above all, to at once assume the recumbent posture should any pain develop in the parts. Cases vary widely in the disposition of the seton; in some this comes away within a fortnight, leaving an ulcer that continues open until the hemorrhoidal tumor disappears; in others, it remains until all the pathological products have been absorbed, and then drops out. In the cases in which I have tested this method I have been well satisfied with the result. It is worthy of trial in cases in which the patient cannot abandon his calling during treatment; in such cases the additional time required is of little consequence compared with the advantage of being able to keep at work while being treated.

If the seton sloughs out and the opening heals with some of the tumor still remaining, a new seton is to be passed, just as if none had ever been introduced. It takes from five to nine weeks to cure an average case by this method.

**Pneumonitis.**—The object of this paper is not to question the almost universal orthodoxy of our leading medical authors as to the local or constitutional theory of this disease, nor to a great extent the theory of self-limitation, but only to present a treatment, new as combined, so far as I know, which in my hands has led me to doubt the theory of self-limitation. I have seen much written concerning ergot and aconite cutting short the attack of pneumonia when used separately. I have given each a fair trial, with but little encouragement. So I concluded to try them combined. I have never been discouraged since in the attempt to cut short this fever. To verify, I will give a clear case of pneumonia, which, I claim, must have been cut short. I was called the 20th of February last to see one R. G., male, 23 years old, of sanguine temperament, good history, etc. He complained of oppression and pain in right side; said he was taken on the night of the 18th with a chill, followed by chilly sensation and pain in breast. Had taken pills and some quinine and Dover's powders. When I saw him his face was flushed, headache, restless, quick pulse, 130 to the minute, respiration increased, temperature 103° F., short cough with sputa slightly colored. Physical examination revealed congestion in lower lobe of right lung, entire; respiratory murmur completely absent. Prescribed

℞ Tinct. aconiti radicis..............................gtts. xxxij.

Fluid ext. ergot......................................ʒ jv.

Potassii brom.........................................ʒ j.

Aquae purae, q. s. to make.......................℥ ij.

Sig. Teaspoonful every hour till four doses were taken, then every two hours till I called again.

21st. Found patient much quieter, much less oppression in breathing, cough not so frequent, sputa more rusty, not very abundant, temperature 100°, pulse 95, lung sound dull with little decrease in boundary. Ordered same treatment continued.

22d. Found patient much better. Temperature 99° F., pulse 80, cough occasionally; lung clearing up, dullness only in small space near center of lower lobe; feels nearly well. Ordered

℞ Tinct. gelsemii.......................................ʒ iij.

Spts. etheris nitrosi.................................ʒ jv.

Aquae purae, q. s. ad...............................℥ ij.

Dose, teaspoonful as above.

23d. Found patient feeling well; no pain; respiration, temperature and pulse normal. Dismissed patient.

The above I claim to be a better success in shortening the run of this fever than we can expect from a treatment of either separately, and far better and more pleasant than the old line of treatment.*Dr. Porter, Michigan Med. News.*

**Resuscitation of a Still Born Infant nearly an Hour after Birth.**—The child was placed in a sitting posture upon blankets before the fire. My hand was placed behind the head and thorax of the child, and its body leaned backward so that it rested upon this hand. The hands of the child were carried as far as possible above its head by my left hand. By this the ribs and shoulders were raised, while the head was thrown backward, thus expanding the thorax and drawing air into the lungs.

The second movement was to lower the arms of the child so that they fell by its side, while my hand, still retaining those of the child in its grasp, rested against the front of the child's thorax and head.

The third movement was to lean the child forward and press suddenly downward upon its shoulders, at the same time that the hand in front pressed the ribs inward.

This method, which is somewhat difficult to describe, but easy to carry out, caused the first certain expulsion of air from the child's lungs (and a consequent refilling of them), for the air could be heard bubbling out through the nose and mouth of the child. Then the lungs were refilled by the first movement. Still this respiration was entirely involuntary on the part of the child, and ten minutes' persistent efforts by this method gave no signs of returning animation.

Nearly three-quarters of an hour had now elapsed since the birth of the child, and meantime the child's skin began to feel cold and clammy. On account of the coldness of the child I determined to use external heat in conjunction with artificial respiration. I called for a deep bowl partly filled with water, at a temperature of about 112°. I placed the child in the bowl in a sitting posture, so that the water came up to the child's diaphragm when its body was in what I have called the 'first position' in the last method of artificial respiration.

Then the artificial respiration was kept up according to this method, while the child's body was partly immersed in hot water. After a few minutes I was astonished at the child showing a little color in its cheeks, and then making its first spasmodic effort at inspiration. Fully five minutes elapsed before it made another gasp, but gradually its efforts became more frequent, and at the end of an hour and three quarters from the time of its birth, I ventured to cease artificial respiration. The child was then breathing rapidly with a shallow, gasping respiration. I ordered it to be rolled in blankets, without being dressed, and was placed in bed with its mother. Two hours later its breathing was stronger, but still shallow. The following day it appeared as strong as any child, and since then has grown rapidly.—*Dr. Forest, in N. Y. Med. Record.*

**Gelsemium in Facial Neuralgia.**—A certain school of practitioners place great dependence on gelsemium as a therapeutic agent, and notwithstanding the suspicion which certain practitioners of another school would cast upon it, there exists abundant testimony to its value in properly selected cases. The National Dispensatory, by Stille and Maisch, lately issued, speaks of it as "one of those too numerous remedies which have been used in a great variety of sthenic febrile diseases upon no better ground than their power of lowering the pulse and depressing the nervous system. Incalculable mischief has been caused by them, and more is likely to be occasioned by gelsemium in febrile affections." Such testimony from such a source is not calculated to predispose one in favor of this article; but when it is known that Professor Stille condemns salicylic acid much more roundly, and accuses it of "incalculable mischief," that the Dispensatory ascribes to grindelia robusta and grindelia squarrosa similar therapeutic properties, and that of all the drugs introduced to the profession within the past few years it regards jaborandi as alone entitled to favor, the force of its objection to gelsemium will be materially weakened.

I have no doubt that I could with safety appeal to the readers of the News for testimony in favor of gelsemium as an antipyretic, but it is not the purpose of this note to argue its general merits; I wish merely to report the following case in testimony of its efficacy in facial neuralgia. I had in my practice a case of this aggravating affection, which had resisted all previous treatment. Relief from pain was only secured by morphia, and this relief was but temporary. The patient was fast becoming addicted to the opium-habit, when I put him on ten-drop doses of fluid extract of gelsemium every three hours. This was two weeks ago, and to-day he came to thank me for the wonderful relief it brought him. I had used the medicine in cases of a similar nature, and with such success as to lead me to regard it as almost infallible.—DR. BURGESS *in Michigan Medical News.*

**Suction Operation for Cataract.**—The Medical Bulletin contains a satisfactory case of this operation at the hands of Dr. R. J. Levis. The Doctor says:

I have had before you during this course a large number of cases of cataract, and nearly all were of the senile or hard variety, but this woman seems to be affected with a cataract of soft consistence. You will remember that the senile form has a more or less striated appearance, while this has a uniform opaque look. From the age of the patient and the appearance of the opacity I think the lens is soft and fluid in the present case, and shall treat it accordingly.

In the other cases of cataract we removed the lens by extraction, after having made a corneal incision and performed iridectomy, but here we shall operate by the suction method. The woman being very nervous we shall administer ethyl bromide by which, as you now see, anæsthesia is produced in three minutes, with only two drachms. I insert a spring speculum between the lids, make an incision in the cornea, introduce a needle to cut up the capsule or use the same knife, and employing the suction apparatus, evacuate the contents of the capsule.

The suction apparatus consists of a delicate rubber tube about ten inches in length, having at one extremity a glass mouth piece and at the other a small delicate metal canula. With a cataract knife I incise the cornea and anterior capsule of lens; then inserting the end of the canula into the anterior chamber, I draw the lens material, by suction produced by my mouth, out into the glass tube, placed near the canula that we may see what is being done. The suction, performed by placing the glass bulb in the mouth, must be carefully adjusted, or damage may result from catching the iris in the canula, thus causing iritis secondarily. The operation is performed and you see how very simple it is. She now sees my hand and can readily count my fingers. Semilunar patches of rubber plaster are placed over the lids of each eye to prevent opening the eyes; and the case is treated as after the operation of extraction, by using atropia locally and keeping the patient in a dark room.

**Tarsal Ophthalmia.**—A case of tarsal ophthalmia is so reported by Dr. R. J. Levis in the Medical Bulletin:

This patient has tarsal ophthalmia, or inflammation of the margins of the eyelids; the condition is sometimes designated ciliary blepharitis. It is an affection of the meibomian glands and the surrounding connective tissue, due to the ducts becoming occluded, by which a low form of inflammation is set up, that soon becomes chronic. The edges of the lids are swollen and painful, and the entire lid is red in color, which redness also extends to the conjunctiva beneath. This condition of lids is frequently found in persons of bad hygienic surroundings, and in very many instances depends especially on accommodative fatigue due to hypermetropia or astigmatism. In the treatment of the affection all causative conditions should be removed, and stimulating applications made to the lids. The best of this kind is water, as hot as the hand can bear, say about 118°, applied to the eye two or three times daily by means of cloths. This acts by checking the secretion of the glands and thus lessens the inflammation. The amorphous yellow oxide of mercury, ten grains to the ounce of ointment, would also answer a very excellent purpose, applied to the lids while keeping the eyes closed. He shall therefore be ordered to bathe the eyes in hot water for five minutes every morning and evening; and to apply three times a day an ointment of the yellow oxide of mercury of the strength mentioned. It is better to give about two drachms of the ointment than a whole ounce, which may become rancid before being used. If he does not improve greatly in a few days it would be proper to examine his eyes for hypermetropia.

**Skin Grafting.**—Dr. R. J. Levis says in Medical Bulletin:

This boy some time ago sustained a lacerated and crushed wound of the hand, which was caught by a cog-wheel. The injury was so considerable that the question of amputation was raised at one time during the early part of the treatment. The parts have partially cicatrized, but there is now a large ulcer upon the wrist which is healing so slowly that it will be proper to employ skin grafting for the purpose of expediting the cure. The granulations have a healthy appearance, and it is probable that grafts will readily start new centres of cicatrization. The operation is performed in this manner: A small sewing needle is thrust under the cuticle of the arm, and made to lift up a minute portion of the upper layers of the skin; this is then clipped off with the scissors, and placed upon the granulations covering the ulcerated surface. It can be done without the occurrence of bleeding and gives very little pain.

The grafts should be placed with their lower surface presenting to the surface of the ulcer. A few days after an operation of this kind, cicatrization begins from these grafts and spreads over the denuded surface, forming little islands of cicatricial tissue. Subsequently these coalesce, and the whole ulcer is healed. In this case twelve grafts have been deposited on different places. No dressing is applied until the lymph and secretions have glued the grafts in position; then carbolized zinc ointment is applied with care, in order to avoid brushing them away.

**Results of Experiments with Iridin and Euonymin on Man.**—Although we must leave to our medical brethren the task of testing on the human subject the effects of baptisin, sanguinarin, phytolaccin, hydrastin, etc., it will doubtless be of service if we here recount our experience of the use of iridin and euonymin. We have found four grains of iridin—made into a pill with a confection of roses, and taken at bed-time—a certain remedy for biliousness. It produces no disagreeable sensations, and, on awaking in the morning, the yellow tongue is found to be clean, and the headache and malaise gone. As iridin, though a powerful hepatic, is not a powerful intestinal stimuiant, it is well to give in the morning an ordinary mild saline aperient, such as Pullna water or some other. But iridin, though an agreeable remedy at the time, has a somewhat depressing effect, and it probably should not be taken oftener than once a week or so. Euonymin is an hepatic stimulant in man as in the dog. Two grains of it, made into a pill with confection of roses, and taken at night, seem to be as efficient a remedy for biliousness as iridin. If the dose be not too great, it leaves no depression. As it is a feeble intestinal stimulant, it is well to follow it in the morning by a dose of Pullna water or other saline aperient. I have been much struck with the success of euonymin in functional hepatic derangement in several persons who had tried nearly all the commonly used cholagogues with varying and often limited success. I have no doubt that, in consequence of our experiments, euonymin will come to be a universally employed stimulant.—*Ex.*

**New Dressing for Superficial Incised Wounds.**—The principle involved in the dressing is a very simple one—a single ligature acting as a lace, and finding fastenings not in the quivering flesh but in plaster on each side of the wound, in which it can readily glide, *laces* the lips of the wound into firm and nice apposition, as the sides of a shoe or corset are laced together. The plaster is prepared for the dressing by being cut into two pieces of sufficient width and length, and are accurately and firmly attached on each side of the wound close to its edges. The edges of plaster adjacent to the wound have previously been furnished with a row of metallic buttons or studs about one-fourth of an inch apart, and securely fastened to the plaster so as to stand up and allow the lace to be attached in a groove which encircles each stud. If the plasters thus prepared have been put in position so that the little studs upon each side are opposed by the spaces intervening between the studs, the lace can be started at one end of the wound and tied at the other, and tightened or loosened at pleasure.

Hooks such as are used on ladies' dresses would answer where the studs could not be had. These I used and found to answer a very good purpose. They should be set back on the plaster far enough to prevent abrasion or chafing of the skin by them.—*Dr. Cowan in Louisville Medical News.*

**Don't Blow your Nose.**—The Medical Herald says it is wrong for one to blow his nose—wrong, not so much because it isn't nice, but because it is dangerous; it jeopardizes the tympanum and irritates the Schneiderian membrane. The epithelium of the latter "is so arranged that blowing the nose detaches these delicate cells just as the scales of a fish would be displaced by forcibly blowing under them. The proper thing to do when the nose becomes suddenly obstructed from swelling in the membrane is to hold the alæ close to the septum and presently snuff strongly, so as to carry the augmented secretion into the throat. In cases of hyperesthesia of the nasal membrane, inhale through the nose and exhale through the mouth. Professor Donders lays great stress upon this method of curing an acute catarrh without medicine." It puzzles one to understand how it is possible to "snuff strongly" with the alæ held close to the septum; but even if such an act were possible, and the substitute for nose-blowing were according to physiological rule, we should prefer the risks of the old-fashioned method to the snuffing and hawking of the new.—*Michigan Medical News.*

**Venesection.**—Dr. Broadbent read the paper of the evening, says the Medical Press and Circular, at the late meeting of Harveian Society of London, on venesection. He pointed out that the fluctuations of medical practice were unfortunate, though some of them were warranted.

Bleeding, from being practiced indiscriminately, has fallen into disuse, though it is often of striking power and is accompanied by very little risk. It does not, in inflammatory affections, strike at the inflammation directly, but lowers the blood pressure in the arteries. When an aneurism is threatening life, bleeding will relieve the tension within its walls, and thus is useful as a palliative. In over-distension of the right heart bleeding is very useful. In those cases of pneumonia where the patient becomes pale, gasps for breath, and is unable to lie down, where the heart beats violently while the pulse is small, then bleeding is indicated, and as the blood flows the pulse improves. He had not bled in bronchitis. In chronic bronchitis with emphysema it did little good. In aortic valvular disease it was not called for. In mitral regurgitation digitalis was to be preferred; but in mitral stenosis the question of bleeding often arose, especially with liver pulsation. Dr. Broadbent then examined at some length the causes of high arterial tension when bleeding is indicated. When the pulse is full between the beats and feels like a tendon, then bleeding is indicated, as in uremic convulsions, in convulsions at times without uremia, in scarlatinal albuminuria, in pregnancy, etc. It was also useful in amenorrhœa in plethoric individuals.

The two main indications for venesection are first, distension of the right ventricle, and second, high arterial tension.

**Bismuth Snuff.**—Dr. Ferrier, in the Lancet, suggests a bismuth snuff for cold in the head. He states that he began to suffer one evening with symptoms of cold in the head—irritation of the nostrils, sneezing, watering of the eyes and commencing flow of the mucus secretion. Having some trisnitrate of bismuth at hand, he took repeated pinches of it in the form of snuff, inhaling it strongly, so as to carry it well into the interior of the nostrils; in a short time the tickling in the nostrils and sneezing ceased, and the next morning all traces of cold had completely disappeared.

But as bismuth by itself is rather heavy and not easily inhaled, and it being also necessary that it should form a coating on the mucous membrane, Dr. Ferrier says it is best to combine it with pulv. acaciæ, which renders the bulk larger and the powder more easily inhalable, while the secretion of the nostrils causes the formation of an adherent mucilaginous coating—of itself a great sedative of an irritated surface, and the sedative effect is greatly strengthened by the addition of a small quantity of hydrochlorate of morphia.—*Journal of Materia Medica.*

**Rubeola in a Pregnant and Puerperal Woman.**—Nelson records the following case:

December 7, 1875, he was called to attend S. D——, two weeks before her expected confinement. The children of the family in which she was living had all been ill with measles, and she had been very freely exposed. As they were convalescing she was taken with the usual premonitory symptoms, and after two and a half days the characteristic rash appeared. The third day of the eruption she was delivered of a healthy boy, the labor not being more than ordinarily severe for a primipara. She made a good recovery. No unusual symptoms were present during the puerperal state other than those directly due to the rubeola. The child was nursed from the first day, the mother having an abundance of milk. The infant remained free from any symptoms of measles, although other children in the house to which the mother had been removed, on the fifth day after confinement, contracted the measles from her.—*St. Louis Courier of Medicine.*

**A Plea for Oleomargarin as a Food Product.**—Representatives of the oleomargarin industry appeared before the House Committee on Agriculture and Manufactures on March 10th, in opposition to any legislation injuriously affecting their product. They claim that oleomargarin is identified with butter; that both the real butter and oleomargarin butter are simply animal fat, and the difference in the process of manufacture makes no difference in the substance. They stated that the factory in New York is now making 40,000 pounds of oleomargarin butter per day, and that there are eleven such factories in Baltimore, Louisville, Chicago and other cities; that the exports of oleomargarin oils from the port of New York alone amounts to 5,000 tierces per month. They protest against any discriminating legislation on the ground that their product is a general food-product, pure and wholesome in itself, and a fit substitute for butter.—*N. Y. Med. Rec.*

**Sweet Cassava Root.**—Sweet cassava root (*Janipha Manihot*) is a native of Brazil, but now acclimated in Florida. When boiled it furnishes a good substitute for the potato; a very beautiful starch can be prepared from it by the usual method, and a very fine article of glucose can be obtained by boiling with dilute sulphuric acid.—*Amer. Journal of Pharmacy.*

**Guarana.**—Dr. Mueller, in Eclectic Medical Journal, recommends guarana in cases like the following. Most frequently the patient will be a female, who, by frequent child-bearing, mental and physical over-exertion, has exhausted the system; she is pale, circulation weak, in fact all the processes of life are feebly executed; the stomach is in a fair condition; even minor disturbances will occasion a spell of headache (mainly frontal, but passing to vertex) of sickening character; efforts to do mental labor will increase the existing trouble. This phenomenon is relieved by refreshing sleep, if such can be obtained. To all appearances there seems to exist an anasarcal state of the cerebrum. Here I prescribe:

℞ Guarana........................................................ ʒj.

Simple syrup.................................................. ℥j.

One teaspoonful every ten to fifteen minutes until relieved, and I am confident that such will follow.

Guarana is a cerebral stimulant of great power; this I am satisfied of, and hence I do not prescribe it in headache the results of colds or determination of blood to the brain, nor will it relieve the condition depending upon a foul stomach and constipated bowels.

**Another Antidote to Arsenic.**—Dr. McCaw, a Canadian physician, suggests the following formula as one not generally known for an antidote to arsenic, and claims for it preference over all others for two reasons, namely, because it forms the surest antidote, and because the ingredients are always accessible.

℞ Tincture of chloride of iron.......................... ʒj

Bicarbonate of soda, or potash...................... ʒj

Tepid water....................................a teacupful.

These are mixed. The sesquioxide of iron is immediately formed in a solution of chloride of sodium.

The mixture may be given almost *ad libitum.*

**Pruritus Ani.**—Wear a piece of cotton wool, of the size of a walnut or larger, at the anus; a few shreds of the wool should be inserted inside the sphincter, and this will be sufficient to retain the whole in its place. A fresh piece must be used after each evacuation. After two years' experience, I can speak most highly of this way of relieving the intolerable nuisance of the pruritus; so long as I wear it I am quite comfortable. For about twelve years I had been a martyr to the complaint. Plugging the nostrils with cotton wool is recommended as a certain cure for paroxysmal sneezing.—*Brit. Med. Journal.*

**Suppression of Menses.**—Dr. Goodell says:

When the suppression comes on suddenly, from cold or exposure while in the midst of the menses, and is accompanied by severe lumbar pains, we should place the patient in a mustard hip-bath, administer Dover's powder, put her to bed, and give her hot drinks to provoke copious diuresis and diaphoresis. Chronic uterine trouble is likely to supervene if we do not act promptly in such cases.—*Louisville Medical News.*

**Dover's Powder for the Night-Sweats of Phthisis.**—The use of a sudorific to check sweating may seem paradoxical, but Dr. Murrell, of the Westminster Hospital, reports having used Dover's powder in fifty-five cases of night-sweating of phthisis, with relief in all cases but five. It also diminishes diarrhœa, eases the cough and insures a good night's rest. He says in conclusion of his very interesting remarks :

"It is often very difficult to make an estimate of the relative value of different modes of treatment in any disease; but I have no doubt that for the night-sweating of phthisis Dover's powder, although it may be inferior to atropia, is far more reliable than oxide of zinc."—*West. Lancet.*

**Balsam of Peru in Pruritus.**—In a communication to the *Deutche Med. Voeh.*, Dr. Auerbach, ot Berlin, states that having, in common with so many other practitioners, found the balsam of Peru a most valuable remedy in itch, he has for some time past treated pruritus by the same substance, and with the greatest success. After the first rubbing in to the part affected great relief is obtained, and in a few days a cure results. He relates a very obstinate case, which, after resisting all kinds of treatment for years, was speedily cured by the balsam.—*Med. Times and Gazette.*

**Incubation Period of Scarlatina.**—The late Dr. Murchison, as a result of his observation and study of the incubation period of scarlet fever, was led to the following conclusions:

1. The duration of the incubation stage may be only a few hours.
2. Probably in a large proportion of cases it does not exceed forty-eight hours.
3. It very rarely exceeds seven days.
4. Consequently, a person who has been exposed to scarlet fever, and does not sicken after a week's quarantine, may be pronounced safe.—*Clinical Soc. Transactions.*

**About Anæsthetics.**—Dr. Yandell says, in Louisville Medical News :

Whether the bromide of ethyl is better than chloroform or ether for prolonged operations is an undetermined question. All anæsthetics are dangerous, but for brief operations I believe it is likely to become popular. In odor it is infinitely less disagreeable than ether, much less so than chloroform, and produces anæsthesia more rapidly than either of these, though less rapidly than nitrous oxide.

**An Improved Nitrate of Silver Caustic.**—A writer in the Medical Times and Gazette, Dr. Sawostizki, has called the attention of the Moscow Surgical Society to an improvement in the preparation of sticks of nitrate of silver. It consists in melting together five parts of nitrate of silver with one part of nitrate of lead, forming an argentum plumbo-nitricum. Sticks formed of this are preferable to those of the ordinary nitrate, as they are not easily broken, and can be pointed, just like a lead pencil.—*Medical and Surgical Reporter.*

